# Sirtuin 3 Activation by Honokiol Decreases Unilateral Ureteral Obstruction-Induced Renal Inflammation and Fibrosis via Regulation of Mitochondrial Dynamics and the Renal NF-κB-TGF-β1/Smad Signaling Pathway

**DOI:** 10.3390/ijms21020402

**Published:** 2020-01-08

**Authors:** Yi Quan, Woong Park, Jixiu Jin, Won Kim, Sung Kwang Park, Kyung Pyo Kang

**Affiliations:** 1 Department of Internal Medicine, Research Institute of Clinical Medicine, Jeonbuk National University Medical School, Jeonju 54907, Korea; quanyi0428@163.com (Y.Q.); mnipw@hanmail.net (W.P.); gilsoo1215@gmail.com (J.J.); kwon@jbnu.ac.kr (W.K.); 2 Biomedical Research Institute, Jeonbuk National University Hospital, Jeonju 54907, Korea

**Keywords:** kidney fibrosis, inflammation, myofibroblast activation, extracellular matrix, Sirtuin 3, mitochondrial dynamics

## Abstract

Renal fibrosis is a common feature of all progressive chronic kidney diseases. Sirtuin 3 (SIRT3) is one of the mitochondrial sirtuins, and plays a role in the regulation of mitochondrial biogenesis, oxidative stress, fatty acid metabolism, and aging. Recently, honokiol (HKL), as a pharmaceutical SIRT3 activator, has been observed to have a protective effect against pressure overload-induced cardiac hypertrophy by increasing SIRT3 activity. In this study, we investigated whether HKL, as a SIRT3 activator, also has protective effects against unilateral ureteral obstruction (UUO)-induced renal tubulointerstitial fibrosis through SIRT3-dependent regulation of mitochondrial dynamics and the nuclear factor-κB (NF-κB)/transforming growth factor-β1 (TGF-β1)/Smad signaling pathway. We found that HKL decreased the UUO-induced increase in tubular injury and extracellular matrix (ECM) deposition in mice. HKL also decreased myofibroblast activation and proliferation in UUO kidneys and NRK-49F cells. Finally, we showed that HKL treatment decreased UUO-induced mitochondrial fission and promoted mitochondrial fusion through SIRT3-dependent effects. In conclusion, activation of SIRT3 via HKL treatment might have beneficial effects on UUO-induced renal fibrosis through SIRT3-dependent regulation of mitochondrial dynamics and the NF-κB/TGF-β1/Smad signaling pathway.

## 1. Introduction

End-stage renal disease (ESRD), requiring renal replacement therapy in the form of dialysis or transplantation, is a major burden for health care, not only in developing countries but in developed countries as well [1]. The incidence and prevalence of chronic kidney disease (CKD) are gradually rising, and mortality and costs remain high even when cases are properly managed [2]. Therefore, prevention and proper management of progressive renal failure is an important therapeutic aim for solving global health problems.

Renal fibrosis, a common feature of all progressive chronic kidney diseases, causes the formation of irreversible scars and renal dysfunction [3]. It can lead to progressive renal failure and finally require renal replacement therapy. The process of renal fibrosis consists of activation of innate and adaptive immune systems, the release of inflammatory and immune mediators, inflammatory cell recruitment and infiltration, persistent myofibroblast activation, and extracellular matrix (ECM) accumulation [3,4]. As part of the process of fibrogenesis, the transforming growth factor (TGF)-β signaling pathway is known to play a key role through the regulation of both anti-inflammatory and profibrotic activity [4].

Honokiol (2-(4-hydroxy-3-prop-2-enyl-phenyl)-4-prop-2-enyl-phenol, HKL) is one of the polyphenols isolated from the seed or bark of the magnolia tree [5]. HKL has various pharmacological effects, including an anti-inflammatory effect through regulation of the nuclear factor-κB (NF-κB) signaling pathway [6], an anti-tumor effect, and an anti-angiogenic effect [7], and acts as a scavenger of reactive oxygen species through NADPH oxidase (NOX)-mediated oxidative stress [8]. HKL also has a protective effect on renal diseases such as acute ischemia–reperfusion injury [9], toxic renal injury [10], and chronic tubulointerstitial fibrosis [11]. These findings suggest that HKL has cytoprotective properties against various noxious stimulations.

Sirtuin 3 (SIRT3) is one of the mitochondrial sirtuins that play a role in the regulation of mitochondrial biogenesis, oxidative stress, fatty acid metabolism, and aging [12,13,14]. SIRT3 has been reported to have a protective effect against cardiac hypertrophy and interstitial fibrosis by augmenting antioxidant defense mechanisms [15]. We also reported that the absence of SIRT3 enhances cisplatin-induced renal inflammation and tubular apoptosis [16]. In aged mice, the SIRT3/TGF-β1 interaction plays an important role in the pathophysiology of pulmonary fibrosis [17]. Recently, HKL, known as a pharmaceutical SIRT3 activator, has been found to block pressure overload-induced cardiac hypertrophy by increasing SIRT3 activity [18]. Therefore, modulation of SIRT3 activity might attenuate the stress-induced organ fibrotic process.

Mitochondria have a crucial role in cellular energy production as a powerhouse that maintains the vitality of the human body. These intracellular organelles are also actively involved in calcium homeostasis, regulation of innate immunity, programmed cell death, and stem cell pluripotency [19,20]. There are multiple pieces of evidence for mitochondrial dysfunction in CKD progression, but a mechanistic dissection of mitochondrial dysfunction in CKD remains elusive [19]. Mitochondrial dynamics modulate the generation of pro-inflammatory mediators such as NF-kB and mitogen-activated protein kinase (MAPK) in activated microglial cells [21]. A recent study suggested that mitochondrial SIRT3-dependent activation of optic atrophy 1(OPA1) contributes to maintain mitochondrial networking and protect cardiomyocytes during cellular stress conditions [22]. Therefore, modulation of mitochondrial homeostasis might be a novel therapeutic target in the treatment of kidney disease.

In this study, we evaluated the protective effect of HKL on unilateral ureteral obstruction (UUO)-induced renal tubulointerstitial fibrosis and its mechanism. Our data show that HKL decreases renal fibrosis through regulation of the NF-κB/TGF-β1/Smad signaling pathway. HKL also decreases UUO-induced mitochondrial fission through the regulation of SIRT3 expression.

## 2. Results

### 2.1. *HKL* Decreases UUO-Induced Renal Tubular Injury and Fibrosis

**Figure 1:**
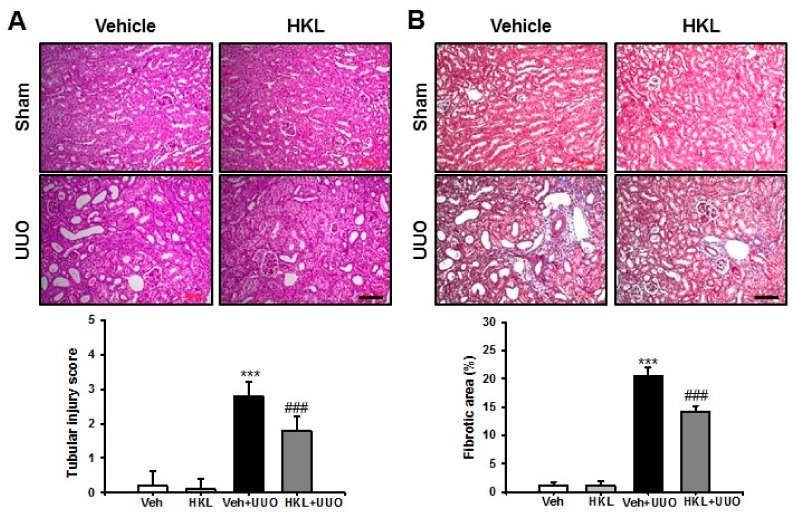
Effect of honokiol on unilateral ureteral obstruction (UUO)-induced renal tubular injury and fibrosis. Representative sections of kidneys from sham- and UUO-operated mice treated with vehicle (Veh) or honokiol (HKL) were stained with (**A**) Periodic acid-Schiff stain (PAS) and (**B**) Masson’s trichrome (MTC). Scale bar = 100 μm. The bar graph shows semi-quantitative scoring of tubular injury by PAS, area fractions (%) of tubulointerstitial fibrosis, and degree of interstitial collagen deposition stained by MTC in the sham and UUO-operated kidneys. Ten randomly chosen, non-overlapping fields were quantified (*n* = 10 per group). Data are expressed as mean ± SD. *** *p* < 0.001 versus Veh or HKL; ### *p* < 0.001 versus UUO treatment with Veh; Sham, sham-operated mice; UUO, unilateral ureteral obstruction; Veh, vehicle; HKL, honokiol.

To evaluate the protective effect of HKL on renal fibrosis, we used the mouse UUO model for induction of renal tubular injury and fibrosis with or without treatment of HKL, and kidney sections were examined after Periodic acid-Schiff stain (PAS) and Masson’s trichrome (MTC) staining. After ten days of ureteral obstruction, UUO kidneys from vehicle-treated mice showed an increase in tubular dilatation, inflammatory cell infiltration, and tubulointerstitial fibrosis, compared with sham-operated kidneys treated with either vehicle or HKL. HKL-treated UUO kidneys showed a decrease in UUO-induced tubular dilatation, inflammatory cell infiltration, and tubulointerstitial fibrosis, compared with vehicle-treated UUO kidneys (Figure 1A,B). These data suggest that HKL has a protective effect on UUO-induced tubular injury and fibrosis.

### 2.2. *HKL* Decreases UUO-Induced Renal Fibroblast Activation and Extracellular Matrix Deposition

**Figure 2:**
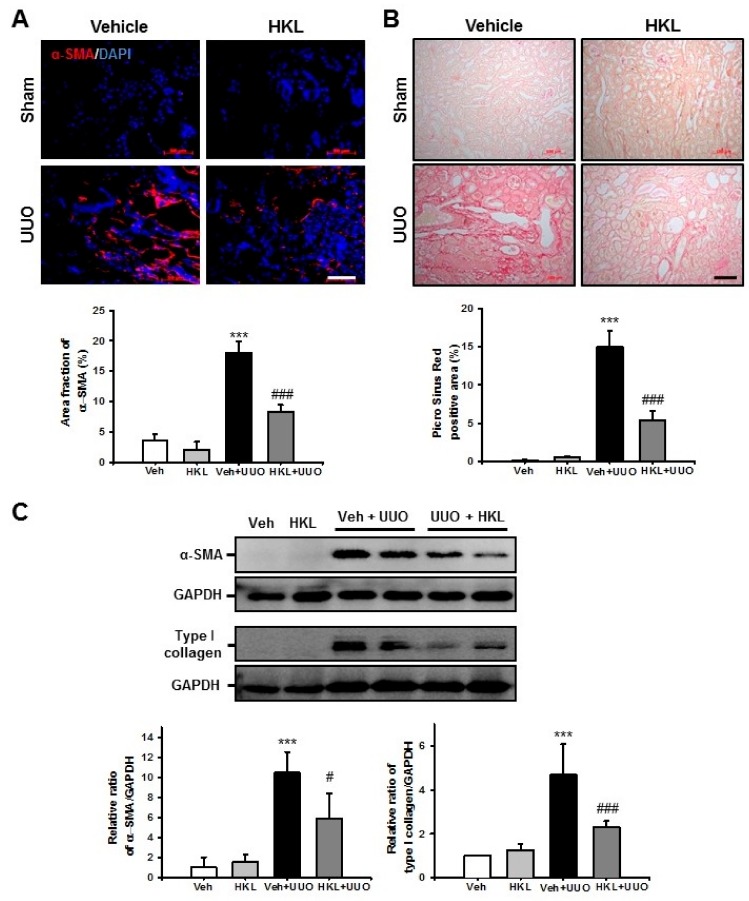
Effect of honokiol on unilateral ureteral obstruction (UUO)-induced myofibroblast activation and extracellular matrix deposition. (**A**) Representative sections of kidneys from sham- and UUO-operated mice treated with Vehicle (Veh) or honokiol (HKL) were immunofluorescence-stained with α-smooth muscle actin (α-SMA) (red). The nucleus was stained by DAPI (blue). Scale bar = 50 μm. The bar graph shows area fractions of α-SMA (%) in the sham and UUO kidneys from ten randomly chosen, non-overlapping fields at a magnification of 400× (*n* = 10 per group). (**B**) The sections were stained with Picro Sirius Red. Scale bar = 100 μm. The bar chart shows Picro Sirius Red (+) areas (%) in the sham and UUO kidneys from ten randomly chosen, non-overlapping fields at a magnification of 200× (*n* = 10 per group). (**C**) Representative Western blot analysis of α-SMA and type I collagen expression in the kidneys from sham- and UUO-operated mice treated with Veh or HKL. The bar graph shows the densitometric quantification presented as the relative ratio of each protein to GAPDH. The relative ratio measured in the kidneys from sham-operated mice treated with Veh is arbitrarily presented as 1. Data are expressed as mean ± SD. *** *p* < 0.001 versus Veh or HKL; # *p* < 0.05 and ### *p* < 0.001 versus UUO treatment with Veh. Sham, sham-operated mice; UUO, unilateral ureteral obstruction; Veh, vehicle; HKL, honokiol; α-SMA, α-smooth muscle actin; DAPI, 4’,6-diamidino-2-phenylindole; GAPDH, glyceraldehyde 3-phosphate dehydrogenase.

Activation of renal fibroblasts and extracellular matrix deposition is a hallmark of renal fibrosis [23]. Therefore, we evaluated renal fibroblast activation after ureteral obstruction. Ten days after UUO surgery, the α-smooth muscle actin (α-SMA)-positive area fraction was increased compared with the sham-operated kidney. HKL-treated UUO kidneys had a significantly decreased α-SMA-positive area compared with vehicle-treated UUO kidneys (Figure 2A). We also assessed α-SMA expression by Western blot analysis in UUO kidneys with or without HKL treatment. UUO kidneys had significantly increased α-SMA expression compared with sham-operated kidneys with vehicle or HKL treatment. However, HKL treatment significantly decreased the UUO-induced increase of α-SMA expression (Figure 2C). To address extracellular matrix deposition after ureteral obstruction, we performed Picro Sirius Red staining. After ureteral obstruction, there was an increase in Picro Sirius Red (+) areas in vehicle-treated UUO kidneys, as compared with the sham-operated kidneys with either vehicle or HKL treatment. However, there was a significant decrease in Picro Sirius red (+) areas in HKL-treated UUO kidneys compared with vehicle-treated UUO kidneys (Figure 2B). We also evaluated type I collagen expression by Western blot in UUO kidneys. Type I collagen expression was increased in vehicle-treated UUO kidneys compared with sham-operated kidneys. However, the UUO-induced increase of type I collagen expression was significantly decreased by HKL treatment (Figure 2C). These data suggest that HKL decreases UUO-induced extracellular matrix deposition in the kidney.

### 2.3. *HKL* Decreases UUO-Induced Renal Inflammation through Regulation of The *NF-κB* Signaling Pathway

**Figure 3:**
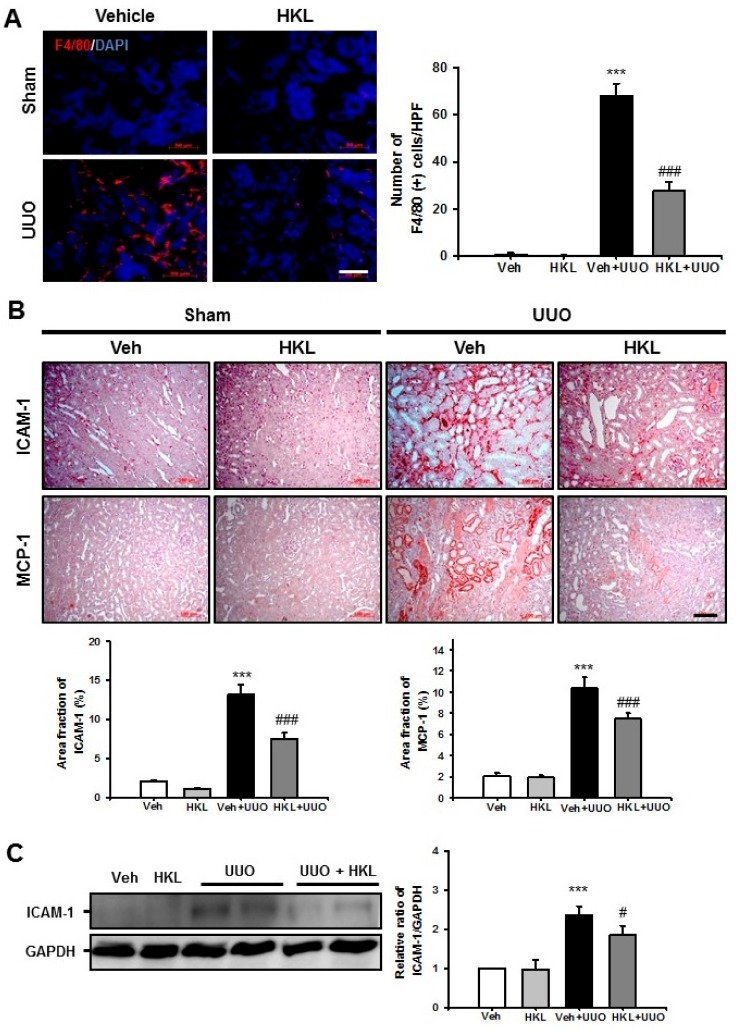
Effect of honokiol on unilateral ureteral obstruction (UUO)-induced renal inflammation. (**A**) Representative sections of kidneys from sham- and UUO-operated mice treated with vehicle (Veh) or honokiol (HKL) were immunofluorescence-stained with F4/80 (red). The nucleus was stained with DAPI (blue). Scale bar = 50 μm. The bar graph shows the number of F4/80-positive cells (%) in the sham and UUO kidneys from ten randomly chosen, non-overlapping fields at a magnification of 400× (*n* = 10 per group). (**B**) Representative sections of kidneys from sham- and UUO-operated mice treated with Veh or HKL were stained with ICAM-1 and MCP-1. Scale bar = 100 μm. The bar graph shows area fractions (%) of ICAM-1 and MCP-1 in the sham- and UUO-operated kidneys from ten randomly chosen, non-overlapping fields at a magnification of 400× (*n* = 10 per group). (**C**) Representative Western blot analysis of ICAM-1 protein expression in the kidneys from sham- and UUO-operated mice treated with Veh or HKL. The bar graph shows the densitometric quantification presented as the relative ratio of each protein to GAPDH. The relative ratio measured in the kidneys from sham-operated mice treated with Veh is arbitrarily presented as 1. Data are expressed as mean ± SD. *** *p* < 0.001 versus Veh or HKL; # *p* < 0.05 and ### *p* < 0.001 versus UUO treatment with Veh. Sham, sham-operated mice; UUO, unilateral ureteral obstruction; Veh, vehicle; HKL, honokiol; ICAM-1, intercellular adhesion molecule-1; MCP-1, monocyte chemoattractant protein-1; DAPI, 4’,6-diamidino-2-phenylindole; GAPDH, glyceraldehyde 3-phosphate dehydrogenase.

After ureteral obstruction, tubulointerstitial inflammation, including macrophage infiltration and increased expression of cell adhesion molecules, is one of the most important findings for the renal fibrosis process. Therefore, we evaluated F4/80 (+) macrophage infiltration after UUO surgery with vehicle or HKL treatment. The number of F4/80 (+) macrophages was significantly increased in vehicle-treated UUO kidneys. HKL treatment significantly decreased the UUO-induced increase of F4/80 (+) macrophage infiltration in tubulointerstitial areas (Figure 3A). We also evaluated intercellular adhesion molecule (ICAM)-1 and monocyte chemoattractant protein (MCP)-1 expression in UUO kidneys with vehicle or HKL treatment by immunohistochemical analyses. After UUO surgery, ICAM-1 and MCP-1 expression both increased in tubulointerstitial areas. HKL treatment significantly decreased the UUO-induced increase of ICAM-1 and MCP-1 expression (Figure 3B). We also evaluated ICAM-1 expression by Western blot analysis. Consistent with immunohistochemical staining, HKL treatment significantly decreased the UUO-induced increase of ICAM-1 expression (Figure 3C).

**Figure 4:**
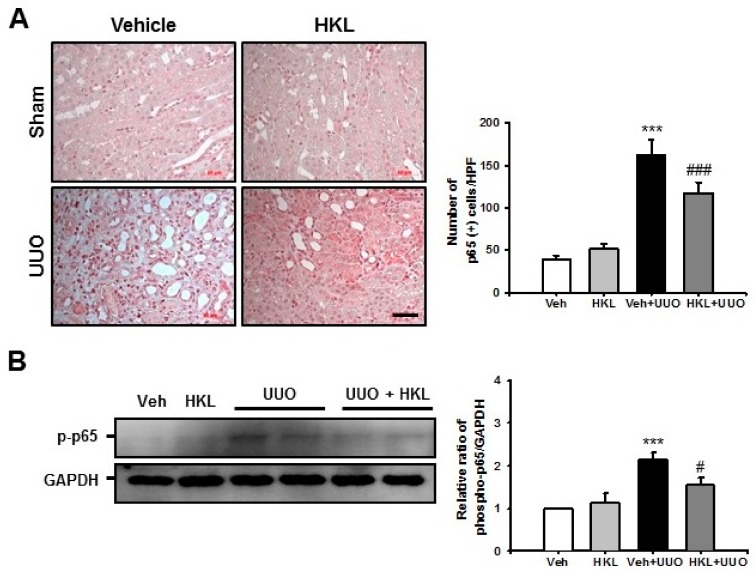
Effect of honokiol on nuclear factor-κB (NF-κB) signaling pathway after ureteral obstruction. (**A**) Representative sections of kidneys from sham- and unilateral ureteral obstruction (UUO)-operated mice treated with vehicle (Veh) or honokiol (HKL) were stained with p65. Scale bar = 50 μm. The bar graph shows the number of nuclear p65 (+) cells in the sham- and UUO-operated kidneys from ten randomly chosen, non-overlapping fields at a magnification of 400× (*n* = 10 per group). (**B**) Representative Western blot analysis of phospho-p65 protein expression in the kidneys from sham- and UUO-operated mice treated with Veh or HKL. The bar graph shows the densitometric quantification presented as the relative ratio of each protein to GAPDH. The relative ratio measured in the kidneys from sham-operated mice treated with Veh is arbitrarily presented as 1. Data are expressed as mean ± SD. *** *p* < 0.001 versus Veh or HKL; # *p* < 0.05 and ### *p* < 0.001 versus UUO treatment with Veh. Sham, sham-operated mice; UUO, unilateral ureteral obstruction; Veh, vehicle; HKL, honokiol; GAPDH, glyceraldehyde 3-phosphate dehydrogenase.

For evaluation of the NF-κB signaling pathway, we performed immunohistochemical staining with p65 after ureteral obstruction with vehicle or HKL treatment. Vehicle-treated UUO kidneys showed an increasing number of nuclear p65 (+) cells compared with the sham kidney. However, HKL treatment significantly decreased the number of nuclear p65 (+) cells in UUO kidneys (Figure 4A). To confirm the involvement of the NF-κB signaling pathway, we also performed Western blot analysis for phosphorylation of p65 in UUO kidneys with vehicle or HKL treatment. Vehicle-treated UUO kidneys showed an increase of phospho-p65 expression compared with sham-operated kidneys. However, HKL treatment decreased the UUO-induced increases in phospho-p65 expression (Figure 4B).

### 2.4. *HKL* Regulates UUO-Induced Changes of Mitochondrial Dynamics through *SIRT3* Activation

**Figure 5:**
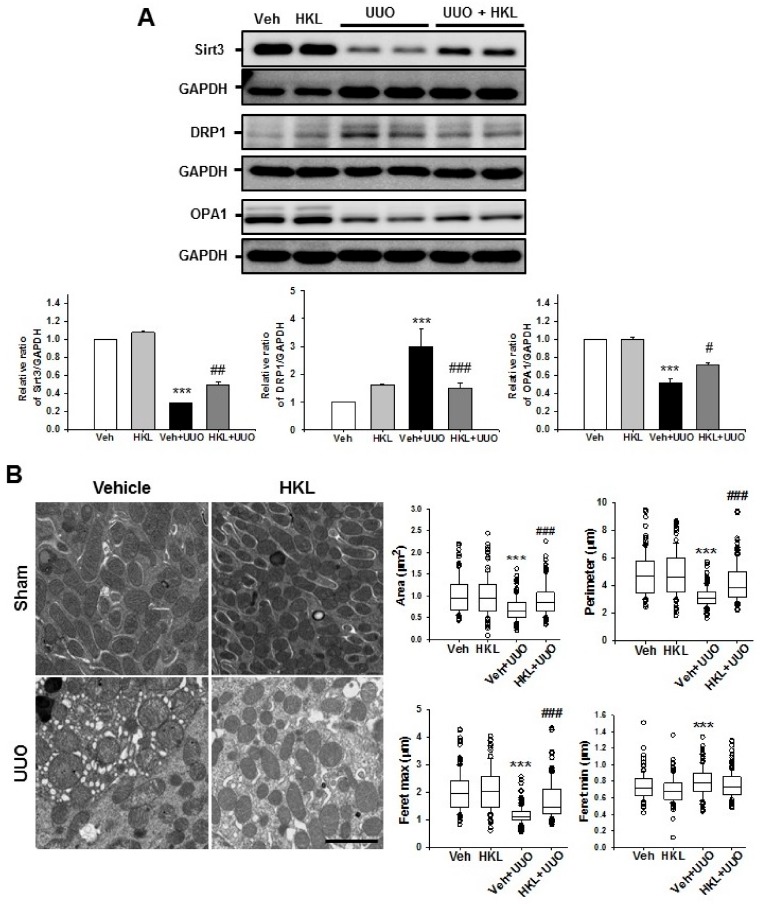
Effect of honokiol on unilateral ureteral obstruction (UUO)-induced Sirtuin 3 (SIRT3) expression and mitochondrial dynamics. (**A**) Representative Western blot analysis of SIRT3, dynamic-related protein (DRP)-1, and optic atrophy 1 (OPA)-1 protein expression from sham- and UUO-operated mice treated with vehicle (Veh) or honokiol (HKL). The bar graph shows the densitometric quantification presented as the relative ratio of each protein to GAPDH. The relative ratio measured in the kidneys from sham-operated mice treated with Veh is arbitrarily presented as 1. Data are expressed as mean ± SD. (**B**) Representative electron micrographs of kidneys from sham- and UUO-operated mice treated with Veh or HKL and their quantification of mitochondrial contour measurements. Scale bar = 2 μm. Data are expressed as mean ± SD of mitochondrial contour measurements from six to eight proximal tubules per mouse (*n* = 10 per group). *** *p* < 0.001 versus Veh or HKL; # *p* < 0.05, ## *p* < 0.01 and ### *p* < 0.001 versus UUO treatment with Veh. Sham, sham-operated mice; UUO, unilateral ureteral obstruction; Veh, vehicle; HKL, honokiol; DRP-1, dynamic-related protein-1; OPA-1, optic atrophy 1; Sirtuin 3, SIRT3.

Mitochondria have an important role in maintaining cellular integrity in both physiologic and non-physiologic, stressful conditions [24]. To address changes in mitochondrial dynamics in UUO-induced renal fibrosis, we evaluated the expression of mitochondrial Sirt3 and mitochondrial dynamin-related protein 1 (DRP1) and optic atrophy 1 (OPA1) expression after ureteral obstruction with vehicle or HKL treatment. Vehicle-treated UUO kidneys showed decreased mitochondrial Sirt3 expression. HKL treatment recovered the UUO-induced decrease of Sirt3 expression (Figure 5A). Mitochondrial fragmentation occurs in both normal physiologic conditions and cellular dysfunctions [24]. During mitochondrial fission, DRP1 is recruited to the mitochondrial outer membrane [19]. Therefore, we evaluated DRP1 expression by Western blot analysis after ureteral obstruction with vehicle or HKL treatment. Vehicle-treated UUO kidneys showed increased DRP1 expression compared with sham-operated kidneys. However, HKL treatment decreased the UUO-induced increase of DRP1 expression (Figure 5A). We also evaluated the expression of mitochondrial fusion protein, OPA1, which is involved in mitochondrial inner membrane fusion and cristae stabilization [19]. OPA1 expression was decreased after ureteral obstruction. However, HKL treatment recovered the UUO-induced decrease of OPA1 expression (Figure 5A).

To determine mitochondrial morphology in vivo, both sham and UUO kidneys were collected for ultrastructural analyses by electron microscopy after ureteral obstruction with vehicle or HKL treatment. Mitochondria from vehicle-treated UUO kidneys were shorter and rounder than those from sham-operated kidneys. Mitochondria from HKL-treated UUO kidneys preserved the usual mitochondrial shape, i.e., larger, longer, and less circular compared with mitochondria from vehicle-treated UUO kidneys (Figure 5B, Table 1). These data suggest that HKL treatment prevents UUO-induced mitochondrial fragmentation by upregulation of mitochondrial Sirt3 expression.

### 2.5. *HKL* Decreases *TGF-β1*-Induced Renal Fibroblast Proliferation and Migration in NRK49F Cells

**Figure 6:**
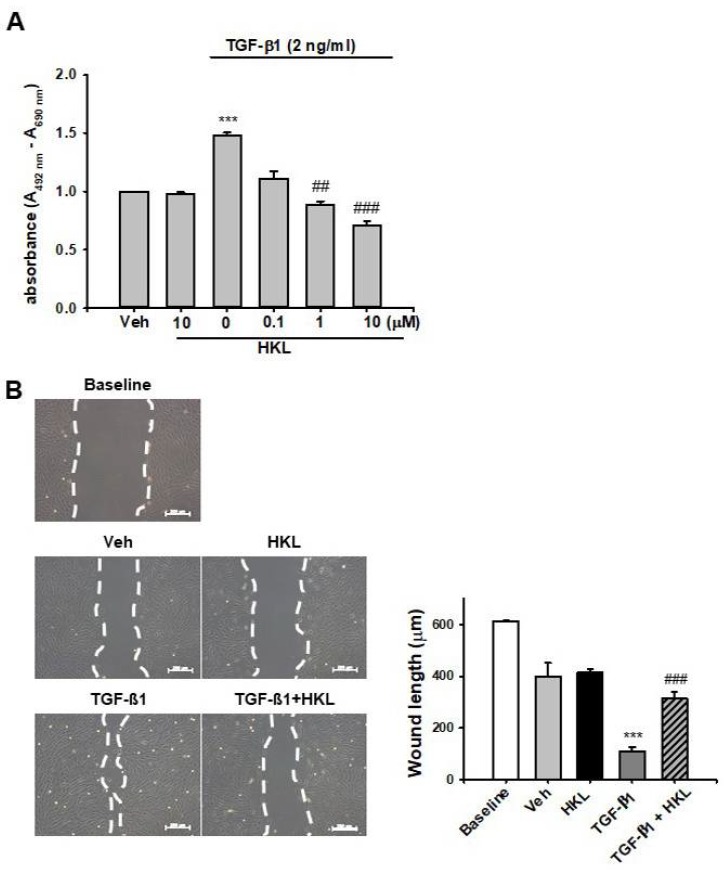
Effect of honokiol on TGF-β1-induced fibroblast proliferation and cell migration in NRK-49F cells (**A**) NRK-49F cells were treated with vehicle (Veh) or transforming growth factor-β1 (TGF-β1, 2 ng/mL), with or without honokiol (HKL) at indicated doses (0.1, 1, and 10 μM). After 24 h of treatment, cell proliferation was measured by XTT assay. Data are expressed as mean ± SD for three independent experiments in triplicates. *** *p* < 0.001 versus Veh or HKL; ## *p* < 0.01, ### *p* < 0.001 versus TGF-β1 (2 ng/mL). (**B**) Representative phase-contrast images for NRK-49F cells after wound healing assay. The phase-contrast images of migration of NRK-49F cells into a scratch area were obtained after treatment with either Veh or TGF-β1 (2 ng/mL), with or without HKL (10 μM), at 0 or 24 h after wounding. The bar graph shows the average length by which the gap between the NRK-49F cells closed over 0 or 24 h after treatment with Veh, TGF-β1, and/or HKL. *** *p* < 0.001 versus Veh or HKL; ### *p* < 0.001 versus TGF-β1. Bar = 200 μm. Veh, vehicle; HKL, honokiol; TGF-β1, transforming growth factor-β1.

To address the protective mechanism of HKL on UUO-induced renal fibrosis, we evaluated TGF-β1-induced renal fibroblast proliferation and migration in vitro using NRK49F cells. Treatment with TGF-β1 increased renal fibroblast proliferation about 1.5-fold compared to vehicle-treated cells. HKL treatment significantly decreased the TGF-β1-induced increase in renal fibroblast proliferation in a dose-dependent manner (Figure 6A). We also evaluated cell migration using a wound healing assay. TGF-β1-treated NRK49F cells were more migrated compared to baseline or vehicle-treated cells. HKL treatment prevents TGF-β1-induced increased cell migration (Figure 6B). These data suggest that HKL treatment regulates TGF-β1-induced renal fibroblast proliferation and migration in vitro.

### 2.6. *HKL* Decreases *TGF-β1*-Induced Renal Fibroblast Activation by Regulation of *TGF-β1/Smad* Signaling Pathway in NRK49F Cells

**Figure 7:**
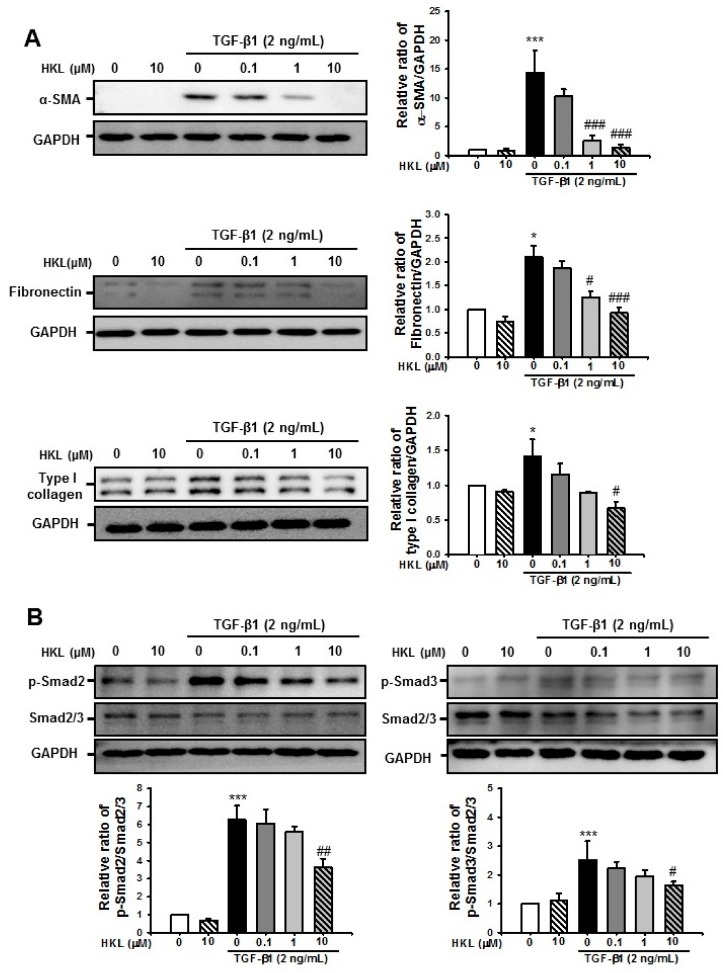
Effect of honokiol on transforming growth factor-β1 (TGF-β1)-induced fibroblast activation and extracellular matrix protein expression by regulation of the TGF-β1/Smad signaling pathway in NRK-49F cells (**A**) Representative Western blot for α-SMA, fibronectin, and type I collagen from NRK-49F cells treated with vehicle (Veh) or TGF-β1 (2 ng/mL), with or without honokiol (HKL) at indicated doses (0.1, 1, and 10 μM). Treatment with TGF-β1 (2 ng/mL) for 24 h increased the expression of fibrotic markers. The expression of α-smooth muscle actin (α-SMA), fibronectin, and type I collagen decreased after HKL treatment in a dose-dependent manner. The bar graph shows the densitometric quantification presented as the relative ratio of each protein to GAPDH. Data are presented as mean ± SD. (**B**) Representative Western blot for phospho-Smad2 and phospho-Smad3 expression from NRK-49F cells treated with vehicle (Veh) or TGF-β1 (2 ng/mL), with or without honokiol (HKL) at indicated doses (0.1, 1, and 10 μM). TGF-β1 (2 ng/mL) treatment for 30 min increased the expression of phospho-Smad2 and phospho-Smad3. Measurement of phospho-Smad2 and phospho-Smad3 protein expression of densitometry is presented as the relative ratio to Smad2/3 protein expression. The bar graph shows the densitometric quantification presented as the relative ratio of each protein to Smad2/3. Data are represented as mean ± SD. * *p* < 0.05 and *** *p* < 0.001 versus Veh or HKL; # *p* < 0.05, ## *p* < 0.01 and ### *p* < 0.001 versus TGF-β1 treatment. Veh, vehicle; HKL, honokiol; TGF-β1, transforming growth factor-β1; α-SMA, α-smooth muscle actin.

To address whether HKL regulates TGF-β1-induced myofibroblast activation and ECM production, we evaluated TGF-β1-induced α-SMA, fibronectin, and type I collagen expression with or without HKL treatment in NRK-49F cells. After 24 h of stimulation, TGF-β1 (2 ng/mL) significantly increased α-SMA, fibronectin, and type I collagen expression. However, HKL treatment significantly decreased the TGF-β1-induced increase of α-SMA, fibronectin, and type I collagen expression in a dose-dependent manner (Figure 7A).

We further evaluated the effect of HKL on the TGF-β1/Smad signaling pathway in NRK-49F cells. Our previous work showed that phosphorylation of Smad2 and Smad3 were peaked at 30~60 min after TGF-β1 stimulation in NRK-49F cells [25]. After 30 min of stimulation, TGF-β1 (2 ng/mL) increased levels of phospho-Smad2 and Smad3 in NRK-49F cells. However, HKL treatment significantly decreased the TGF-β1-induced phosphorylation of Smad2 and Smad3 proteins in a dose-dependent manner (Figure 7B). These data suggested that HKL modulates TGF-β1-induced renal fibroblast activation through the TGF-β1/Smad signaling pathway.

## 3. Discussion

The normal wound repair process occurs in a highly regulated sequence of steps such as inflammation, new tissue formation, and remodeling [26,27]. These wound healing processes also share similar mechanisms with fibrosis [26]. However, if this process becomes pathologic, organ fibrosis and failure are ensured. Therefore, researchers are trying to determine novel therapeutic targets that promote normal wound repair. Our current work focuses on the role of Sirt3 activation in UUO-induced renal fibrosis. Treatment with HKL, which is a SIRT3 activator, decreases UUO-induced renal inflammation, myofibroblast activation and proliferation, and ECM deposition through regulation of the NF-kB and TGF-β/Smad signaling pathway. More interestingly, HKL reverses UUO-induced decrease of SIRT3 expression and regulates UUO-induced mitochondrial fusion and fission in in vivo experiments. Therefore, activation of SIRT3 by HKL may have therapeutic potential through promoting mitochondrial dynamics as well as regulating inflammation and myofibroblast activation in UUO-induced renal tubulointerstitial fibrosis.

Inflammatory reactions such as macrophage infiltration and pro-inflammatory cytokine production are major pathophysiologic mechanisms of renal interstitial fibrosis following ureteral obstruction [28]. Chronic and persistent inflammation causes progressive renal injury, finally leading to permanent damage to the renal parenchyma [29]. In addition, chronic inhibition of the NF-kB signaling pathway attenuates renal damage and inflammation in the 5/6 renal ablation model [30]. Therefore, the regulation of the renal inflammatory response might play an important role in the treatment of UUO-induced renal injury. Our results support the idea that treatment of HKL decreases macrophage infiltration, pro-inflammatory cytokine production, and cell adhesion molecule expression in UUO kidneys through regulation of NF-kB p65 phosphorylation and nuclear translocation.

Activation of myofibroblasts is a hallmark of renal fibrosis, which emerge de novo in the interstitium of diseased kidneys [31]. Injured renal tubules and infiltrated inflammatory cells produce profibrotic factors, which results in myofibroblast activation through paracrine or autocrine mechanisms [32]. Activated myofibroblasts produce various types of ECM such as collagen, fibronectins, elastin, fibrillins, and proteoglycans, which contribute to fibrosis [33]. Compared to quiescent fibroblasts, myofibroblasts have abundant rough endoplasmic reticulum and are typically surrounded by collagen fibers [32,33]. Therefore, targeting myofibroblast activation is one of the novel therapeutic options for treatment of renal fibrosis. Our data shows that HKL treatment decreases TGF-β1-induced NRK-49F cell proliferation and migration. Furthermore, HKL treatment decreases the UUO-induced increase of ECM expression. These protective effects of HKL on renal fibrosis might be associated with decreases in myofibroblast activation through regulation of the TGF-β1/Smad signaling pathway.

Morphologic change in mitochondria during cellular injuries, such as mitochondrial fragmentation, is a critical process contributing to renal pathophysiology [34]. TGF-β1 is involved in the pro-fibrogenic process after acute or chronic kidney injury. On the one hand, increases in TGF-β1 in various renal cells directly or indirectly leads to mitochondrial dysfunction [35,36]. Excessive mitochondrial fission or decreased fusion may be related to mitochondrial dysfunction and cellular derangement [37]. Sirt3 is localized in the mitochondrial matrix and regulates the fundamental integrity of cells, including respiratory chain activity, the tricarboxylic acid (TCA) cycle, fatty acid β-oxidation, and the antioxidant pathway [20]. In addition, SIRT3-dependent activation of OPA1 contributes to mitochondrial dynamics such as favoring mitochondrial fusion and cell survival in doxorubicin-induced cardiomyocyte death [22]. Therefore, modulation of SIRT3 activity in diseased kidneys might be a novel therapeutic mechanism for the treatment of acute or chronic kidney disease. In our study, SIRT3 expression was significantly decreased in vehicle-treated UUO kidneys and promotes mitochondrial fission by increasing DRP1 expression. Morphologically, UUO kidneys produced shorter and rounder mitochondria compared to sham-operated kidneys. On the contrary, HKL treatment recovered SIRT3 expression in UUO kidneys and also promoted mitochondrial fusion by increasing OPA1 expression. HKL-treated UUO kidneys produced mitochondria having a longer and more elliptical shape compared to vehicle-treated UUO kidneys. These data support the idea that HKL treatment decreases UUO-induced mitochondrial fragmentation through regulation of Sirt3-dependent mitochondrial dynamics.

The proposed mechanism of SIRT3 activation by HKL might be related to direct interaction between SIRT3 and HKL to promote deacetylation of mitochondrial targets [18]. Our data also shows the effect of HKL to block UUO-induced renal fibrosis through SIRT3-dependent effects. The study further demonstrated that SIRT3 activation by treatment with HKL on UUO kidneys had an anti-inflammatory effect in the regulation of the TGF-β1/Smad signaling pathway to suppress myofibroblast activation and migration and promote mitochondrial fusion.

In conclusion, activation of SIRT3 by HKL treatment might have a beneficial effect on UUO-induced renal fibrosis through SIRT3-dependent regulation of mitochondrial dynamics and the NF-κB/TGF-β1/Smad signaling pathway.

## 4. Materials and Methods

### 4.1. Animal Experiment

The animal experiment protocol was reviewed and approved by the Institutional Animal Care and Use Committee of Jeonbuk National University, Jeonju, Korea (CBNU 2016-19, approved on 29 February 2016). For UUO surgery, 8-week-old C57BL/6 mice weighing 19–22 g (Orient Bio Inc., Seoul, Korea) were used and maintained in a room under controlled temperature (23 ± 1 °C), humidity, and lighting (12 h light/12 h dark cycle), and with free access to water. All of the mice were divided into four groups of 12 mice each: sham with vehicle treatment, UUO with HKL treatment, UUO with vehicle treatment, and UUO with HKL treatment. Honokiol (HKL; Sigma Chemical Co., St Louis, MO, USA) was dissolved in dimethyl sulfoxide (DMSO, 0.05% *v*/*v*), which was used as the vehicle. HKL (5 mg/kg) was administered by daily intraperitoneal injection for 7 d prior to UUO surgery and continued for 10 d after UUO surgery.

Renal fibrosis was induced by UUO operation as described previously [23,25]. In brief, mice were anesthetized via intraperitoneal injection of ketamine (100 mg/kg, Huons, Seoul, Korea) and xylazine (10 mg/kg, Bayer Korea, Seoul, Korea) and placed on a temperature-controlled operating table with body temperature maintained at 37 °C. After a midline incision in the abdomen, the right proximal ureter was exposed and ligated at two separate points using 3–0 black silk. The sham operation was performed using the same method without ligation of the ureter. Ten days after surgery, the obstructed kidney was harvested, prepared for histologic examination, and stored at −80 °C for Western blot analysis.

### 4.2. Histologic Examination

The kidneys were fixed via immersion in 4% paraformaldehyde, dehydrated by washing in a series of increasing ethanol concentrations, and then embedded in paraffin. The block was cut into 5 μm sections and stained with periodic acid-Schiff stain (PAS) and Masson’s trichrome. Immunohistochemical staining was performed as described previously [16,25,38]. Tissue sections were deparaffinized with xylene, rehydrated through graded washes of ethanol in water, and rinsed in pure water. After a heat-induced antigen retrieval process and treatment with the blocking buffer, slides were incubated overnight at 4 °C with either a rabbit anti-mouse monocyte chemoattractant protein-1 (MCP-1; 70R50662; dilution 1:100; Fitzgerald Industries International, Acton, MA, USA) and hamster anti-mouse intercellular adhesion molecule-1 (ICAM-1; 553249; dilution 1:100; BD Biosciences, San Jose, CA, USA), or rabbit anti-mouse NF-κB p65 (sc-8008; dilution 1:100; Santa Cruz Biotechnology, Santa Cruz, CA, USA). The kidney sections were treated with DAKO Chromogen (DAKO Cytomation, Glostrup, Denmark) to visualize immunocomplexes and then counterstained with hematoxylin (Sigma Chemical Co.). For immunofluorescence staining, freshly frozen renal tissues were fixed with 4% paraformaldehyde, permeabilized in 1% Triton X-100, and then incubated with a blocking buffer. The tissue samples were incubated with either a rat anti-mouse F4/80 (14-4801-82; dilution 1:200; eBioscience, San Diego, CA, USA) or a mouse anti-α-smooth muscle actin (α-SMA; dilution 1:200; BD Biosciences, Allschwil, Switzerland). Slides were exposed to Cy3-labeled secondary antibody (Chemicon, Temecula, CA, USA). Nuclear staining was performed using 300 nM 4’, 6-diamidino-2-phenylinole solution for 1 min (DAPI; Molecular Probes; Thermo Fisher Scientific, Inc. Waltham, MA, USA). For morphometric analysis, two observers who were unaware of the origins of samples used a Zeiss Z1 microscope (Carl Zeiss, Göttingen, Germany) to evaluate all slides. The tubular injury was scored at six levels on the basis of the percentage of tubular dilatation, epithelial desquamation, and loss of brush border in ten randomly chosen, non-overlapping fields at a magnification of 200× under a light microscope: 0, none; 0.5, <10%; 1, 10%–25%; 2, 25%–50%; 3, 50%–75%; and 4, >75%. The fibrotic areas and positive areas of ICAM-1 and MCP-1 were measured in ten randomly chosen, non-overlapping fields at a magnification of 200× using ImageJ software (Version 1.47, National Institutes of Health, Bethesda, MD, USA, http://rsb.info.nih.gov/ij). The number of p65-positive tubule cells and F4/80-positive macrophages were counted at a magnification of 400×.

Transmission electron microscopy was performed as previously described [39]. Kidneys were fixed in 0.1 M cacodylate buffer with 2.5% glutaraldehyde. Ultrastructural changes of the renal tubules, especially for the extent of the mitochondrial changes, were examined by Hitach H-7600S transmission electron microscopy (Hitach, Tokyo, Japan). For ultrastructural analyses, all mitochondrial contours within photographed tubules (25–50 mitochondrial contours per tubule) were drawn to obtain an unbiased estimate of mitochondrial morphology using ImageJ software as previously described [24]. Contour measurements (area, perimeter, feret minimum and maximum, roundness, aspect ratio, and shape factor) were automatically calculated using ImageJ software. Feret maximum and minimum correspond to the longest and shortest projections, respectively, of the minimal bounding box (i.e., parallel tangents apposing opposite sides of the profile). The aspect ratio describes the degree of flatness of a contour with values ranging from zero to one, where a circle has a value of one. Roundness values range from zero to one, with a circle having a value of one. The shape factor represents the outline of a contour: a large shape factor has a convoluted outline, while a circle has the smallest shape factor at a value of 3.54. Contour measurements are related as follows:

$$Aspect\;Ratio=\frac{MaxDiameter}{MinDiameter}$$

$$Shape\;factor=\frac{Perimeter}{\sqrt{Area}}$$

$$Roundness=\frac{4\times Area}{\pi \times MaxDiameter^{2}}$$

### 4.3. Picro Sirius Red Stain

For evaluation of the collagen deposition after ureteral obstruction, paraffin-embedded tissue sections were stained with Picro Sirius red [23]. After deparaffinization, sections were hydrated and stained with 0.1% Picro Sirius red solution (Sigma Chemical Co., St Louis, MO, USA) for 1 h. After washing in acidic water, tissue sections were dehydrated and mounted. The Picro Sirius red-positive areas were measured in ten randomly chosen, non-overlapping fields at a magnification of 200× using ImageJ software.

### 4.4. Western Blotting

Western blot analysis was performed as described previously [23]. Primary antibodies to α-SMA (A2547; mouse; dilution 1:1,000; Sigma Chemical Co., St Louis, MO, USA), ICAM-1 (sc-1511; goat; 1:1000) and fibronectin (sc-6953; goat; 1:500) (Santa Cruz Biotechnology, Santa Cruz, CA, USA), type I collagen (1310-01; goat; 1:1000; Southern Biotech, Birmingham, AL, USA), SIRT3 (5490S; rabbit), DRP1 (5391; rabbit), OPA1 (80,471; rabbit), phospho-Smad2 (3101; rabbit) and phospho-Smad3 (9520; rabbit) (1:1000; Cell Signaling Technology, Danvers, MA, USA), and Smad2/3 (07-408; rabbit; 1:1000; EMD Millipore, Billerica, MA, USA) were used. Glyceraldehyde 3-phosphate dehydrogenase (GAPDH; AP0063; rabbit; 1:2000; Bioworld Technology, Inc., St. Louis Park, MA, USA) was used as an internal control. All signals were analyzed by densitometric scanning (LAS-3000, Fuji Film, Tokyo, Japan).

### 4.5. Cell Culture Experiment

In vitro experiments were performed using a rat renal fibroblast cell line (NRK-49F, American Type Culture Collection, Manassas, VA, USA). NRK-49F cells were cultured in Dulbecco’s modified Eagle’s medium with 4 mM L-glutamine adjusted to contain 1.5 g/L sodium bicarbonate and 4.5 g/L glucose supplemented with 5% (*v*/*v*) heat-inactivated fetal bovine serum and antibiotics (100 U/mL penicillin G and 100 μg/mL streptomycin) at 37 °C with 5% CO_2_ in 95% air. To investigate the effect of HKL on myofibroblast activation, extracellular matrix expression, and activation of the TGF-β1/Smad signaling pathway, subconfluent NRK-49F cells were incubated with HKL (0.1, 1 and 10 μM) for 30 min and then stimulated with TGF-β1 (2 ng/mL, Sigma Chemical Co., St Louis, MO, USA) for indicated time periods.

### 4.6. Cell Proliferation Assay

After 24 h treatment with HKL (0.1, 1, and 10 μM) and TGF-β1 (2 ng/mL), the proliferation of NRK-49F cells was determined by a colorimetric assay (Cell Proliferation Kit II, Roche Diagnostics, Mannheim, Germany) according to the manufacturer’s protocol. All experimental values were determined from triplicate wells.

### 4.7. Wound Healing Assay

Subconfluent NRK-49F cells were cultured in six-well dishes. Before treatment with HKL and TGF-β1, dishes were scratched using a sterile 200-μL pipette tip, causing three separate wounds. The cells were incubated with HKL (10 μM) for 30 min and then stimulated with TGF-β1 (2 ng/mL) for 24 h. Wound lengths were measured using the ImageJ program. The wound length at 0 h after scratching was used as the control.

### 4.8. Statistical Analysis

Data were expressed as mean ± S.D. Multiple comparisons were examined for significant differences using analysis of variance (ANOVA), followed by individual comparison with Tukey’s post hoc test, with *p* < 0.05 indicating statistical significance.

## Figures and Tables

**Table 1:** Contour measurements of mitochondria.

Parameters	Sham		UUO	
	Veh	HKL	Veh	HKL
Roundness	0.378 ± 0.171	0.349 ± 0.165	0.718 ± 0.178 ^a,c^	0.485 ± 0.197 ^b,c^
Aspect ratio	3.198 ± 1.376	3.427 ± 1.480	1.509 ± 0.494 ^a,c^	2.532 ± 1.341 ^b,c^
Shape factor	4.793 ± 0.749	4.801 ± 0.694	3.849 ± 0.250 ^a,c^	4.445 ± 0.771 ^b,c^

^a^*p* < 0.05 compared with sham + Veh; ^b^ *p* < 0.05 compared with UUO + Veh; ^c^ *p* < 0.05 compared with sham+HKL. Sham, sham-operated mice; UUO, unilateral ureteral obstruction; Veh, vehicle; HKL, honokiol.

## Abbreviations

α-SMA	α-smooth muscle actin
CKD	Chronic kidney disease
DAPI	4’,6-diamidino-2-phenylindole
DRP-1	Dynamic-related protein-1
ESRD	End-stage renal disease
ECM	Extracellular matrix
GAPDH	Glyceraldehyde 3-phosphate dehydrogenase
HKL	Honokiol
ICAM-1	Intercellular adhesion molecule-1
MAPK	Mitogen-activated protein kinase
MCP-1	Monocyte chemoattractant protein-1
NOX	NADPH oxidase
NF-κB	Nuclear factor-κB
NRK	Normal rat kidney
OPA-1	Optic atrophy 1
TGF-β1	Transforming growth factor-β1
UUO	Unilateral ureteral obstruction
Veh	Vehicle
